# An outbreak following importation of wild poliovirus in Xinjiang Uyghur Autonomous Region, China, 2011

**DOI:** 10.1186/s12879-015-0761-y

**Published:** 2015-01-31

**Authors:** Hai-Bo Wang, Wen-Zhou Yu, Xin-Qi Wang, Fuerhati Wushouer, Jian-Ping Wang, Dong-Yan Wang, Fu-Qiang Cui, Jing-Shan Zheng, Ning Wen, Yi-Xin Ji, Chun-Xiang Fan, Hui-Ling Wang, Gui-Jun Ning, Guo-Hong Huang, Dong-Mei Yan, Qi-Ru Su, Da-Wei Liu, Guo-Min Zhang, Kathleen H Reilly, Jing Ning, Jian-Ping Fu, Sha-Sha Mi, Hui-Ming Luo, Wei-Zhong Yang

**Affiliations:** Chinese Center for Disease Control and Prevention, 27 Nanwei Road, Xicheng District, Beijing, 100050 PR China; Peking University Clinical Research Institute, Xueyuan Rd 38#, Haidian District, Beijing, 100191 PR China; Expanded Programme on Immunization, Xinjiang Uyghur autonomous region Center for Disease Control and Prevention, 138 Jianquanyi Street, Urumqi City, Xinjiang Uyghur autonomous region 830001 PR China; The Center for Disease Control and Prevention of the Xinjiang Production and Construction Corps, 344 Wuxingnanlu Street, Urumqi City, Xinjiang Uyghur autonomous region 830002 PR China; WHO WPRO Regional Polio Reference Laboratory, National Institute for Viral Disease Control and Prevention, Chinese Center for Disease Control and Prevention, 155 Changbai Rd, Changping District, Beijing, 102206 PR China; Independent Consultant, New York City, NY USA

**Keywords:** Wild poliovirus, Importation, Acute flaccid paralysis, Supplementary immunization activities, Serological survey

## Abstract

**Background:**

After more than 10 years without a case of wild poliovirus (WPV) in China, an outbreak occurred in 2011 in Xinjiang Uyghur Autonomous Region.

**Methods:**

Acute flaccid paralysis (AFP) case surveillance was strengthened with epidemiological investigations and specimen collection and serological surveys were conducted among hospitalized patients.

**Results:**

There were 21 WPV cases and 23 clinical compatible polio cases reported. WPV was isolated from 14 contacts of AFP cases and 13 in the healthy population. Incidence of WPV and clinical compatible polio cases were both highest among children <1 years, however, 24/44 (54.5%) polio cases were reported among adults aged 15–39 years.

**Conclusions:**

High coverage of routine immunization should be maintained among children until WPV transmission is globally eradicated. Expansion of AFP case surveillance and use of serologic surveys to estimate population immunity should be conducted rapidly to guide preparedness and response planning for future WPV outbreaks.

## Background

Historically, poliomyelitis had been endemic and widespread in China, with approximately 20,000 paralytic cases reported annually since it was included in the national disease surveillance system in 1953. Interrupting transmission of wild poliovirus (WPV) was an important public health priority of the newly founded People’s Republic of China. By administering oral attenuated poliovirus vaccine (OPV) nationwide, establishing the Expanded Programme on Immunizations in 1978, developing a cold chain system and strengthening regular immunization services, the number of poliomyelitis cases declined dramatically [1,2]. Since 1990, the implementation of Supplementary Immunization Activities (SIAs), resulted in the eradication of indigenous WPV. The last indigenous case of WPV was reported in September 1994 [1]. The Western Pacific Region (WPR), which encompasses China, was certified as polio-free in October 2000 [3].

Despite significant achievements since the launch of the Global Polio Eradication Initiative in 1988, circulation of indigenous WPV continues in three countries (Afghanistan, Nigeria, and Pakistan) in 2012, and many previously polio-free countries have been affected by WPV spread from remaining endemic countries [4-7]. China, which shares border with two of three endemic countries, has experienced three instances of WPV importation: 1995 and 1996 in Yunnan Province [8], and 1999 in Qinghai Province [9,10]. Because of the high quality of the acute flaccid paralysis (AFP) surveillance system and emergency response, WPV importations were detected and responded to in time, without subsequent transmission.

After being polio-free for more than 10 years, on Aug 25, 2011, an outbreak was confirmed in Xinjiang Uyghur Autonomous Region (Xinjiang), China following importation of type I WPV originated from neighboring Pakistan [11,12]. The last indigenous WPV case was reported in 1994 in Xinjiang prior to the outbreak. This report describes the characteristics of the outbreak, findings from serological investigations of healthy populations, and the response based on the national preparedness plan.

## Methods

### Setting and population

Xinjiang is situated in the northwest with 14 prefectures, accounting for one-sixth of the total geographical area of China. It shares 5,600 kilometers of international borders with Mongolia in the northeast, with Russia, Kazakhstan, Kyrgyzstan and Tajikistan in the west, and with Afghanistan, Pakistan and India in the southwest. In 2011 the population was estimated to be 21.8 million with 4.5 million children younger than 15 years of age; the average population density is 13 persons/km^2^, and the annual birth rate is approximately 14.9‰.

Four OPV doses are recommended at ages 2, 3, and 4 months and 4 years. OPV is delivered at specified community health service centers in urban areas or administered by village doctors in rural areas. Since 1990, Xinjiang has conducted two rounds of SIAs with OPV targeting all children ≤4 years old every year regardless of their prior immunization history. Although the reported coverage of routine immunization is >95%, actual coverage is thought to be considerably lower [1,13], as it is difficult to determine the number of target children for immunization due to population mobility and since provincial health departments are reluctant to report low coverage because of performance assessment. This underestimate is reflected in the results of the outbreak study’s serological survey. In 2011, reported measles incidence in Xinjiang was 8.7 cases/100,000 population, 12 times the national average.

### Surveillance for AFP cases

China established the AFP surveillance system in 1993 to support polio eradication according to World Health Organization (WHO) guidelines. County Centers for Disease Control (CDC) staff are responsible for conducting investigations of AFP case which are reported by hospitals within 2 days after being identified and reviewing records of all hospitals in the surveillance system every 10 days. After the WPV outbreak was confirmed, retrospective AFP case finding and “zero daily reporting” (daily reporting regardless of whether AFP cases were found or not) were initiated in hospitals at the township level or above for AFP cases of all age groups in southern Xinjiang (Hotan, Kashgar, Bayinguole, Kezilesukeer and Akesu prefectures) and the provincial capital, Urumqi where there are most of provincial hospitals (patients with serious illness such as paralysis would choose to visit these hospitals), as well as in hospitals at the county level or above for AFP cases younger than15 years of age in other prefectures [14].

An AFP case was defined as a child under 15 years of age presenting with AFP, or as any person at any age with paralytic illness if poliomyelitis was suspected. A WPV case was defined as a person with AFP for whom a stool specimen tested positive for WPV by virology. A clinical compatible polio case was defined as an AFP case who tested negative for WPV on inadequate stool specimens, but was determined to be polio-compatible by the provincial Polio Expert Committee (PEC) of China after the standard 60-day follow-up examination. Persons with AFP who tested negative for poliovirus on adequate stool specimens or who were judged by provincial PEC to not be polio-compatible were defined as non-polio AFP cases. In keeping with definitions used in previous polio outbreaks, those ≥15 years of age were considered adults [15,16].

### Serological surveys

Between August 29^th^ and September 8^th^, 2011, immediately after the confirmation of WPV importation, a serological survey was conducted in southern Xinjiang before the first round of SIAs were conducted. Those who visited hospitals at the county level or above for a blood extraction for reasons not related to the polio investigation were invited to participate. An additional 2-ml blood sample was collected from each subject by venipuncture. Neutralization antibodies against poliovirus serotype 1 (P1), 2 (P2) and 3 (P3) were determined by a microneutralization assay with authentic Sabin strains in accordance with WHO guidelines [17]. A serum sample was considered positive if the neutralization antibody level was present at a dilution ≥1:8.

### Epidemiological investigation and specimen collection

Teams consisted of epidemiologists, clinicians and virologists who investigated each AFP case and reviewed clinical records. The teams also investigated contacts of AFP cases, including people who lived in the same household, neighbors, hospital contacts and classmates, as well as healthy children in the townships where WPV cases were found. Demographic characteristics, immunization records and contact information were investigated, and fecal specimens were collected for contacts and healthy children.

### Isolation and characterization of poliovirus isolates

Stool specimens were forwarded to the provincial polio laboratory where viral isolation was performed on L20B and RD cell cultures, and viral isolates were identified by micro-neutralization assay. Poliovirus isolates are forwarded to the National Polio Laboratory where intratypic differentiation was performed by polymerase chain reaction–restriction fragment–length polymorphism and by enzyme-linked immunosorbent assay. The full VP1 genomic region of WPV isolates was sequenced with an ABI Prism BigDye Terminator Cycle Sequencing Ready Reaction kit and an automated DNA sequencer. Sequence data were compared with those of reference strains (GenBank).

### SIAs

**Figure 1:**
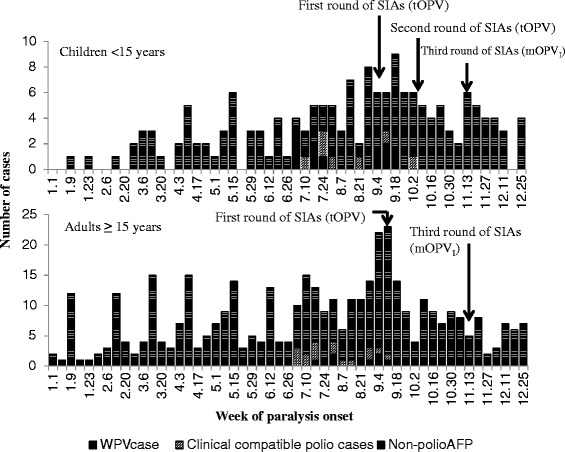
**AFP cases by week of paralysis onset and timing of SIAs in Xinjiang, 2011.** WPV case clinical compatible polio cases non-polio AFP. WPV: wild poliovirus; AFP: acute flaccid paralysis. Note: The first SIAs was conducted among population <40 years of age in Southern Xinjiang, children < 15 years in Urumqi, and children <5 years in other prefectures. The second round of SIAs was conducted targeting children < 15 years in Southern Xinjiang and Urumqi, and children <5 years in other prefectures. The third round of SIAs targeted the same population as the first round of SIAs.

A total of five rounds of SIAs were conducted in Xinjiang: three rounds in 2011 and two rounds in 2012. Three weeks after the virus was isolated, the first SIAs were conducted among children (<15 years in Southern Xinjiang and Urumqi, <5 years in other prefectures) during September 8–12 (Figure 1), followed by adults aged 15–39 years in Southern Xinjiang September 13–26. The second round of SIAs were conducted targeting children (the same as the first round) October 8–12. The third (November 15–22), fourth (March 17–25, 2012) and fifth (April 16–25, 2012) round of SIAs targeted the same children and adults aged 15–39 years in Southern Xinjiang [12].

### Ethical considerations

This study was approved by the Chinese Center for Disease Control and Prevention institutional review board (IRB). Written informed consent was provided by participants or their guardians for participants who were children, after study staffs explained fully to them about the purpose of the study, and the risks and benefits of anonymizing publication.

## Results

### AFP surveillance

**Figure 2:**
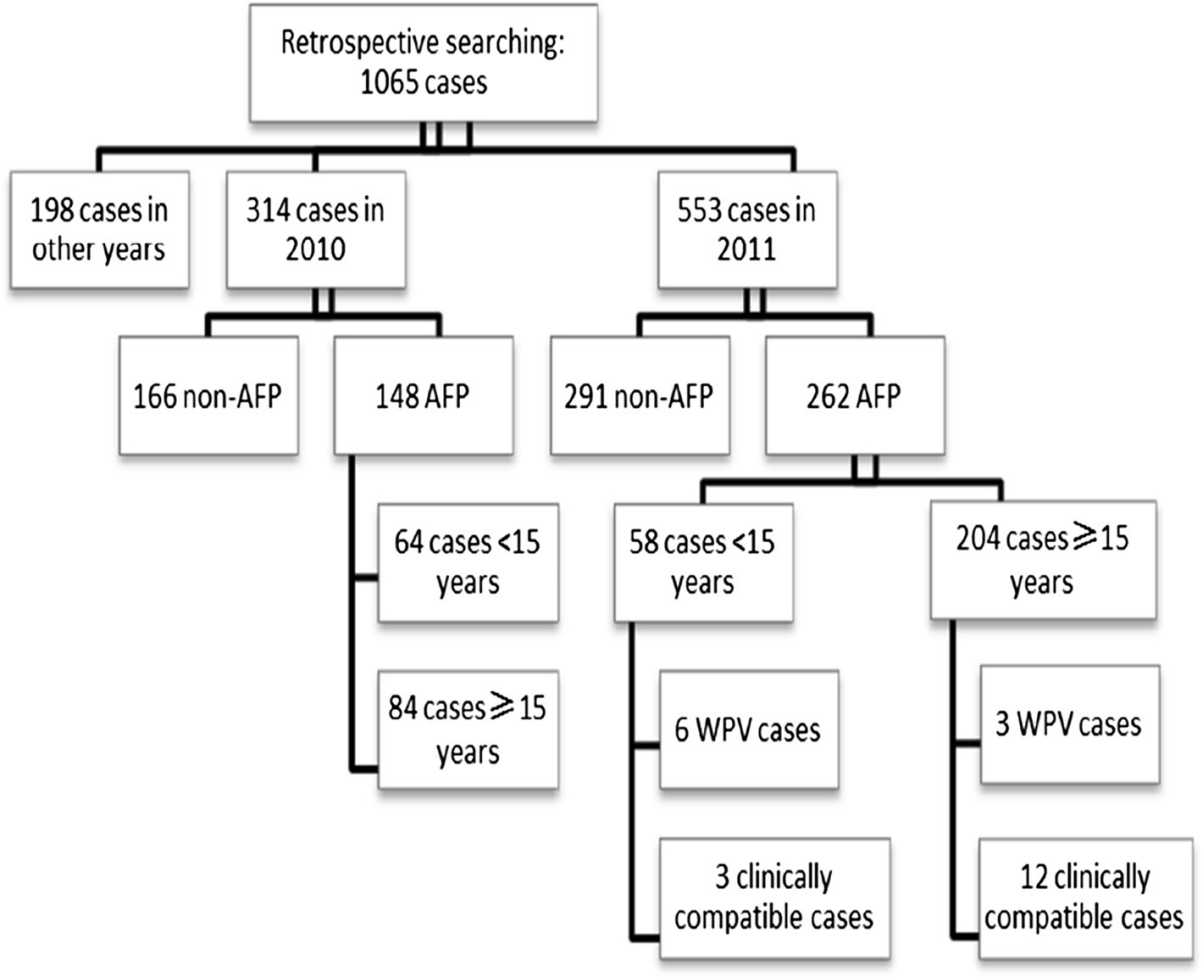
**The illustration of suspected AFP cases in retrospective AFP searching.**

A total of 1065 suspected AFP cases were detected through retrospective searching of AFP cases that had onset of paralysis since January 2010 (Figure 2). Among 410 cases identified as AFP cases, 148 had onset of paralysis in 2010 and 262 had onset of paralysis in 2011 (58 in children and 204 in adults); 161 of these adult cases in 2011 lived in Urumqi and Southern Xinjiang. Of these additional cases detected by retrospective searching, 15 were clinical compatible polio cases and 9 were WPV cases. A total of 578 AFP cases were detected and confirmed in 2011 by combining 219 cases identified retrospectively and 359 cases monitored prospectively in “zero daily reporting”.

148 non-polio AFP cases <15 years of age were reported with the incidence of 3.28 per 100, 000 in 2011, compared with 76 cases with the incidence of 1.63 per 100, 000 in 2010. Of the 148 non-polio AFP cases among children in 2011, 68 had onset of paralysis before the confirmation of outbreak with the incidence of 2.26 per 100, 000, and 80 paralyzed after that with the incidence of 5.32 per 100, 000. Of the 165 reported AFP cases <15 years old, two adequate fecal specimens were collected within 14 days of paralysis onset among 49/79 (62.0%) AFP cases who had onset of paralysis before outbreak confirmation, and among 73/86 (84.9%) AFP cases who had onset of paralysis after outbreak confirmation.

Of 578 AFP cases (165 children <15 years and 413 adults ≥15 years) reported in 2011, 21 (3.6%) were WPV cases and 23 (4.0%) were clinical compatible polio cases. The first WPV case had onset of paralysis on July 3^rd^, and the last WPV cases on October 9^th^, just at the initiation of the second round of SIAs (Figure 1), while the first clinical compatible polio case had onset of paralysis on July 5^th^, and the last case on October 4^th^. The two periods of June 3^rd^ to July 31^st^ and September 4–25 had the greatest number of reported WPV and clinical compatible polio cases.

WPV transmission was limited in four administrative prefectures in southern Xinjiang: Hotan, Kashgar, Bayingolin and Akesu. Incidence of WPV and clinical compatible polio cases were highest among children <1 years (3.46/100,000 and 1.15/100,000 respectively), followed by children aged 1–4 years (Table 1) [12,14]. However, no WPV cases were reported among children aged 5–14 years, and only one clinical compatible polio case was reported among this age group.

**Table 1:** **Incidence rates of WPV and clinical compatible polio cases by demographical characteristics in 2011 in Xinjiang**

**Characteristics**		**WPV and Clinical compatible polio cases** ^**§**^					
		**WPV cases**		**Clinical compatible polio cases**		**Total**	
		**No.**	**Incidence (/100,000)**	**No.**	**Incidence (/100,000)**	**No.**	**Incidence (/100,000)**
Age (Years)	<1	6	3.46	2	1.15	8	4.62
	1–4	4	0.64	4	0.64	8	1.28
	5–14	0	0.00	1	0.07	1	0.07
	15–39	10	0.22	14	0.31	24	0.52
	≥40	1	0.04	2	0.07	3	0.11
Sex	Male	14	0.29	20	0.41	34	0.70
	Female	7	0.15	3	0.06	10	0.21
Prefectures	Hotan	13	0.65	19	0.94	32	1.59
	Kashgar	6	0.15	3	0.08	9	0.23
	Akesu	1	0.04	1	0.04	2	0.08
	Bayingolin	1	0.08	0	0.00	1	0.08
	Total	21	0.22	23	0.24	44	0.46
Xinjiang^¶^		21	0.10	23	0.11	44	0.20

^§^Incidence was calculated based on the population in 4 prefectures where WPV was isolated.

^¶^Incidence was calculated based on the population in Xinjiang.

Genetic sequencing of type I WPV isolated from the index case showed that the strains diverged from the VP1 region of the type I Sabin strain by more than 20%, and were about 99% homologous with one another. WHO confirmed that the outbreak had been caused by WPV imported from Pakistan on the basis of nucleotide sequence data.

### Contacts of AFP cases and healthy population

Stool specimens were collected from 673 contacts of AFP cases, and WPV was isolated from 14 contacts, seven of whom were the contacts of four non-polio AFP cases (in which WPV was isolated from four contacts of one case). Among the four non-polio AFP cases, two adequate specimens were collected from three cases, and two inadequate specimens from one case. WPV was isolated from 3 contacts of 2 clinical compatible polio cases (in which WPV was isolated from 2 contacts of one case). The highest rate of WPV isolation was among the contacts aged <1 year, followed by contacts aged 5–14 years (4.3%). WPV was isolated among 7/340 (2.1%) contacts of AFP cases aged 15–39 years (Table 2). After the first round of SIAs (finished on September 27^th^ for adults), there was only one contact from whom WPV was isolated.

**Table 2:** **The isolation results for the contacts of AFP cases and healthy population**

**Population**	**Characteristics**		**WPV**	**NPEV** ^**§**^	**Vaccine strains**	**Negative**	**Total**
			**N (%)**	**N (%)**	**N (%)**	**N (%)**	
Contacts of AFP cases	The type of AFP cases^∮^	WPV cases	4 (1.4)	0 (0)	4 (1.4)	269 (97.1)	277
		Clinical compatible	3 (7.0)	1 (2.3)	0 (0)	39 (90.7)	43
		polio cases					
		Non-polio AFP cases	7 (2.0)	7 (2.0)	15 (4.3)	324 (91.8)	353
	Age (Years)	<1	1 (4.8)	1 (4.8)	2 (9.5)	17 (80.9)	21
		1–4	0 (0)	2 (2.2)	1 (1.1)	87 (96.7)	90
		5–14	4 (4.3)	1 (1.1)	6 (6.4)	83 (88.3)	94
		15–39	7 (2.1)	3 (0.9)	8 (2.4)	322 (94.7)	340
		≥40	2 (1.6)	1 (0.8)	2 (1.6)	123 (96.1)	128
	Sex	Male	7 (2.4)	5 (1.7)	12 (4.0)	274 (91.9)	298
		Female	7 (1.9)	3 (0.8)	7 (1.9)	358 (95.5)	375
	Districts	Urumqi	0 (0)	0 (0)	1 (1.0)	102 (99.0)	103
		Hotan	8 (3.2)	2 (0.8)	9 (3.5)	235 (92.5)	254
		Kashgar	6 (3.2)	4 (2.1)	5 (2.7)	172 (92.0)	187
		Bayingolin	0 (0)	1 (1.5)	2 (2.9)	65 (95.6)	68
		Aksu	0 (0)	1 (1.9)	2 (3.8)	50 (94.3)	53
		Other prefectures	0 (0)	0 (0)	0 (0)	8 (100.0)	8
	Specimens collection date	Before 9 Sep	8 (2.4)	4 (1.2)	4 (1.2)	321 (95.2)	337
		9 Sep- 27 Sep	5 (2.2)	4 (1.7)	8 (3.4)	216 (92.7)	233
		After 27 Sep	1 (1.0)	0 (0)	7 (6.8)	95 (92.2)	103
	Total		14 (2.1)	8 (1.2)	19 (2.8)	632 (93.9)	673
Healthy population	Age (Years)	<1	0 (0.0)	0 (0.0)	0 (0.0)	12 (100.0)	12
		1–4	4 (4.0)	4 (4.0)	4 (4.0)	89 (88.1)	101
		5–14	8 (3.5)	5 (2.2)	41 (18.1)	172 (76.1)	226
		15–39	1 (1.1)	0 (0.0)	2 (2.2)	90 (96.8)	93
		≥40	0 (0.0)	2 (3.4)	1 (1.7)	56 (94.9)	59
	Districts	Urumqi	0 (0.0)	0 (0.0)	2 (5.9)	32 (94.1)	34
		Kashgar	5 (5.6)	1 (1.1)	2 (2.2)	81 (91)	89
		Hotan	8 (2.5)	9 (2.8)	43 (13.3)	263 (81.4)	323
		Other prefectures	0 (0.0)	1 (2.2)	1 (2.2)	43 (95.6)	45
	Total		13 (2.7)	11 (2.2)	48 (9.8)	419 (85.3)	491

^§^Non-polio enterovirus.

^∮^Four non-polio AFP cases and 2 clinical compatible polio cases should be classified as WPV cases based on International Health Regulations (IHR, 2005) which defined that an AFP case with WPV isolation in stool specimens collected from a close contact of the case is classified as an WPV case.

WPV was also isolated from 13/491 (2.7%) of the healthy population who lived in the same or nearby townships with WPV cases. The highest rate of WPV isolation was among children aged 1–4 years (4.0%), followed by children aged 5–14 years (3.5%). There was no statistically significant difference in the WPV isolation rate between children aged 1–4 years and children aged 5–14 years of age. WPV was isolated among 1/93 (1.1%) healthy adults aged 15–39 years (Table 2). The healthy children infected by WPV were located in Hotan and Kashgar prefectures. A survey was conducted in Beijing among the students from Xinjiang; stool specimens were collected from 671 students and WPV was isolated from three persons without onset of paralysis.

### Serological surveys

Among the 2,611 subjects enrolled, 2,253 (86.3%), 2,283 (87.4%), and 1,989 (76.2%) were seropositive to P1, P2 and P3 respectively at titers ≥1:8. Overall, only 1744 (66.8%) subjects were positive to all the three serotypes: 83.2% in children aged 1–4 years and 79.5% in children aged 5–14 years. We observed a correlation between age group and seropositivity: the antibody seroprevalence was highest in children aged 5–14 years for P1 and P2, and the highest seropositivity was in children aged 1–4 years for P3. For all 3 serotypes of poliovirus, the antibody seroprevalence was lowest in children <1 year of age, and the second lowest antibody seroprevalence was in adults aged 15–39 years. In addition, 94 (3.6%) subjects possessed detectable antibodies against P1 only: 14/198 (7.1%) children aged <1 year, 9/435 (2.1%) children aged 1–4 years, 12/596 (2.0%) children aged 5–14 years, 42/1059 (4.0%) adults aged 15–39 years, and 17/323 (5.3%) adults aged ≥40 years.

## Discussion

This report documents the first introduction of WPV in China since being certified as polio-free in 2000 following the importation from neighboring Pakistan [11]. Surveillance among contacts of AFP cases and healthy populations, as well as serological surveys showed circulation of WPV and gaps in population immunity. Children less than 5 years of age were most likely exposed and vulnerable to the virus, and the large number of susceptible children provided basis for adults infection, conversely, adults who accounted for more than 50% polio cases may be an important source of infection for children. These epidemiological findings and low immunity in young adults justify vigorous interventions. Prompt SIAs among the population <40 years of age in southern Xinjiang and among children in other prefectures within one month of WPV isolation may have prevented further spread of the virus.

Children <5 years of age, particularly children <1 year of age, were most vulnerable as they had the highest WPV incidence, which was also supported by the serological surveys. Both the lowest antibody titers and the lowest seropositive rates were detected among children <1 year of age who could not complete the primary vaccination schedule, as well as 7.1% of children <1 year of age possessed antibody against P1 only, which suggested they were exposed to type I WPV responsible for the outbreak. Only 83.2% children aged 1–4 years of age and 79.5% children aged 5–14 years were seropositive for all three serotypes, which may imply low quality of routine immunization and SIAs in these years that result in a larger susceptible population. The inadequate coverage of routine immunization may be related to ethnic or religious factors, migration, or transit inaccessibility. In southern Xinjiang, most of habitants are Uyghur ethnicity, some of whom have fears about vaccine safety or adhere to religious beliefs, which reject immunizations. Some children who live in poor and remote villages are hard to reach for immunization due to transit inaccessibility. The children missed by routine immunization could also be missed by series of SIAs, although SIAs were conducted every year and reported coverage was >95%.

Serological surveys can be used to gauge underlying gaps in population polio immunity and subsequently guide preparedness and response planning [16,18,19]. A study conducted in 2010 showed that almost all (95.8%) children <15 years of age in Xinjiang were seropositive to type 1 poliovirus, however the study was mainly conducted in Urumqi and Northern Xinjiang where no WPV was isolated [19]. Both high antibody titers and high seropositive rates for poliovirus were found among children aged 5–14 years in which only one clinical compatible polio case was reported, this age group had the lowest incidence [20]. For all 3 serotypes of poliovirus, the antibody seroprevalence was lowest in children <1 year of age, and the second lowest antibody seroprevalence was in adults aged 15–39 years [20]. However, a high rate of WPV isolation was found among children aged 5–14 years of age (3.5% in healthy population and 4.3% in close contacts of AFP cases), which indicated the potential role in WPV transmission for children aged 5–14 years of age, even if only one clinical compatible polio case was reported among this age group.

Commonly, it is expected that higher antibody seroprevalence were for P2 relative to other serotypes of poliovirus [19,21]. In our serologic surveys, antibody seroprevalence of P2 (87.4%) was comparative with P1 (86.3%). Moreover, the higher seroprevalence of P1 relative to P2 was found among children <1 year of age and among adults aged ≥40 years of age, which indicated a pervasive transmission in southern Xinjiang.

Based on the findings, from this 2011 WPV outbreak investigation, the target population for SIAs was expanded to <40 years in southern Xinjiang. The fact that the last WPV case had onset of paralysis on October 9^th^ (the day after the beginning of second round of SIAs), despite enhanced AFP surveillance, suggested that transmission of WPV might have been dramatically reduced by the first round of SIAs. Limited transmission among few unvaccinated individuals seems not to have been enough to result in a clinical case of poliomyelitis. The success of quick restriction of WPV transmission highlights the importance of SIAs for adults in southern Xinjiang. Success of this program could not have been achieved without conducting high quality SIAs. The rapid implementation of SIAs was the result of excellent collaboration between health services, community networks, government and international partner organizations.

Adults aged 18–31 years accounted for almost 50% of polio cases, although with relatively low incidence. In addition to vaccination, another method of protection is past exposure to WPV strains. However, opportunities for unvaccinated populations to acquire natural immunity through WPV exposure have greatly decreased, as transmission of WPV is reduced with increasing immunization coverage since the early 1980s; the coverage was not yet universally high enough at the beginning of routine immunization, and many may also have missed the NIDs that began in 1993 if they were not in the targeted age group; these factors possibly contributed to the increase of the susceptible population and allowed for a large number of polio cases in the 18–31 age group when WPV was imported. Any country with no recent WPV transmission may face similar outbreaks characterized by a large proportion of cases in older age groups [22-28]. An attack rate of 10 per 100, 000 persons were reported among adults aged 19–25 years in Albania in 1996 [23] and 14/19 (74%) confirmed cases were adults aged 15–29 years in Namibia in 2006 [22]. Similarly, the current investigation found 42/1059 (4.0%) adults aged 15–39 years and 17/323 (5.3%) adults aged ≥40 years possessed detectable antibodies against P1 only; this suggests that these adults may have been exposed to the type I strain responsible for the outbreak and high transmission probability. More importantly, these adults were a potential source of WPV for children in their family.

Our study has several limitations. The number of WPV cases may be under-reported based on the Chinese national AFP surveillance guideline. The definition of WPV case in the Chinese national AFP surveillance guideline differs from the case definition of the International Health Regulations (IHR, 2005). Under the IHR, an AFP case with WPV isolation in stool specimens collected from a close contact of the case is classified as an WPV case, whereas isolation results in stool specimens collected from close contacts had no impact on the diagnosis of WPV cases in the current study. The 2 clinical compatible polio cases in our study, from whose contacts WPV was isolated, should be diagnosed as WPV cases based on IHR. Secondly, the serological survey was based on convenience sampling, enrolling participants hospitalized at county-level or above. The seroprevalence of antibody against poliovirus may be over-estimated as the children who are not reached by immunization activities may be less likely to be hospitalized in high level hospitals.

## Conclusions

Until WPV transmission is globally eradicated, the risk of WPV importation exists even in countries which have been certified as polio-free [5,6,29-31]. Therefore, high coverage of routine immunization should be maintained in children until WPV transmission is globally eradicated, in addition, routine vaccination should be reinforced by preventive SIAs in high risk WPV importation areas to avoid the accumulation of susceptible individuals. As experienced by China, the Republic of the Congo [16,18,32], Namibia [22], Cape-Verde [15], Albania [25], and Tajikistan [30], any country with no WPV transmission for a long period of time and relatively low levels of vaccination coverage may encounter similar outbreaks characterized by a large proportion of cases in adults. Based on the evidence of immunity gaps determined by serologic surveys, SIAs should be conducted among adults, if necessary, in future WPV outbreaks. In addition, expansion of AFP case surveillance should be conducted rapidly to fully understand the epidemiological characteristics for future WPV outbreaks. Infection burden among adults should also be considered in future WPV outbreaks, especially for countries without WPV transmission in recent years. Lessons from the WPV outbreak in China can aid in the preparation of prevention and response measures for future outbreaks in other parts of the world.
